# Real-World Challenges of Haplo-Identical Hematopoietic Stem Cell Transplant in a Developing Country: A Single Center Experience

**DOI:** 10.7759/cureus.59744

**Published:** 2024-05-06

**Authors:** Ammad Akram, Mehwish Gilani, Qamar un Nisa Chaudhry, Raheel Iftikhar, Tariq Ghafoor, Mehreen Ali Khan, Memoona Haider, Haider Nisar

**Affiliations:** 1 Clinical Hematology, Armed Forces Bone Marrow Transplant Center, Rawalpindi, PAK; 2 Chemical Pathology, Armed Forces Bone Marrow Transplant Center, Rawalpindi, PAK; 3 Hematology, Armed Forces Bone Marrow Transplant Center, Rawalpindi, PAK; 4 Hematology and Oncology, Armed Forces Bone Marrow Transplant Center, Rawalpindi, PAK; 5 Pediatrics, Armed Forces Bone Marrow Transplant Center, Rawalpindi, PAK

**Keywords:** transplant, stem cell, hematopoietic, haplo-identical, real world

## Abstract

Hematopoietic stem cell transplant (HSCT) is potentially, the sole curative option for many malignant and non-malignant hematological disorders. Finding a human leukocyte antigen (HLA) compatible donor remains one of the limiting factors, hampering the utilization of HSCT. However, the introduction of post-transplant cyclophosphamide (PTCy) has improved the outcomes of haploidentical transplants making it a suitable option for patients lacking HLA-compatible donors. We collected data from 44 patients who underwent haplo-identical allogeneic stem cell transplants at the Armed Forces Bone Marrow Transplant Center/National Institute of Blood and Marrow Transplant (AFBMTC/NIBMT) from the year 2015 to 2022. The diseases were divided into three categories, i.e., bone marrow failure (BMF) syndromes, hematological malignancies (HM) and miscellaneous (Misc) groups. Median age at transplant was 18 (01-39) years. Transplant indications included aplastic anemia (AA) in 21 (47.7%) cases, 15 (34.1%) HM, and eight (18.2%) cases falling in the Misc groups. A maximum number of graft failures occurred in the BMF group; primary graft failure in 07 (33.3%) cases and secondary graft failure in four (19%) cases, (p-value < 0.05). Acute graft versus host disease (aGVHD) grade II-IV occurred in nine (20.5%) cases while chronic graft versus host disease (cGVHD) occurred in 10 (22.7%) cases. Cytomegalovirus (CMV) reactivation was seen in 31 (70.5%) cases. Maximum CMV reactivation was seen in HM group 13 (86.6%) cases, (p-value < 0.05) as compared to BMF (71.4%) and Misc groups (37.5%). Post-transplant cyclophosphamide (PTCy) based regimens, early neutrophil engraftment, and patients with GVHD had better survival outcomes (p-value < 0.05) overall survival (OS), and relapse-free survival (RFS). and GVHD-free relapse-free survival (GFRS) were significantly better in cases with early neutrophil engraftment. OS of the study cohort was 50% while disease-free survival (DFS) and GFRS were 45.5% and 36.4%, respectively.

## Introduction

Hematopoietic stem cell transplant (HSCT) is potentially, the sole curative option for many malignant and non-malignant hematological disorders [1]. With the increasing advancement in diagnostic modalities and improvement in HSCT outcomes, an increasing number of patients are now being considered for HSCT [2]. Finding a human leukocyte antigen (HLA)-compatible donor remains one of the limiting factors, hampering the utilization of HSCT [3]. However, the advent of newer transplant strategies has resulted in a global increase in haplo-identical HSCT. This is evident from the data published by CIBMTR, which shows a rise in haplo HSCT transplants, and it has surpassed Matched Related Donor (MRD) transplants in the year 2021. Similar results are seen in data from the Hematopoietic Cell Transplantation and Cellular Therapy Survey of the European Society for Blood and Marrow Transplantation (EBMT) in 2021, showing an increasing trend in haplo-identical HSCT starting from the year 2010 onwards [4]. The major breakthrough in haploidentical HSCT was achieved by the introduction of post-transplant cyclophosphamide (PTCy). Other strategies such as T-cell depletion with either “mega dose” CD34+ cells or selected α/β T-cell and B-cell depletion, G-CSF-mobilized allografts, and anti-thymocyte globulin (ATG), have also contributed to significant improvements in haplo-HSCT outcomes in recent decades [5-7].

In contrast to the situation in developed countries, haplo HSCT is a challenging treatment option in resource-limited settings. These challenges primarily stem from an underdeveloped healthcare infrastructure, high prevalence of regional Cytomegalovirus (CMV) seropositivity, higher prevalence of multidrug-resistant bugs, lack of newer and more effective antiviral and antifungal agents, non-availability of newer transplant techniques like use of total body irradiation (TBI) in haplo-identical HSCT, T-cell depletion techniques, etc., and chronically malnourished patients at the time of transplant, etc. The prospects are further reduced due to the paucity of specialized supportive care, the cost of newer strategies, the lack of experienced staff, and the non-availability of certain chemotherapeutic and immunosuppressive agents in some cases [8].

This study’s cohort includes a diverse group of patients with various hematological malignancies (HM), bone marrow failure (BMF) syndromes, and immune-mediated disorders, who underwent haploidentical HSCT. While PTCy alone and PTCy + ATG represent potent and promising approaches in haplo-identical HSCT, they are not without various unique challenges in resource-limited settings [9]. The aim of this article was to share our experience of haplo-identical HSCT at a tertiary care center and elaborate on the consequent real-world challenges in resource-limited settings of third-world countries.

## Materials and methods

This study was a retrospective, single-center study conducted at the Armed Forces Bone Marrow Transplant Center/National Institute of Blood and Marrow Transplant (AFBMTC/NIBMT) Rawalpindi, Pakistan. After approval from the Institutional Review Board and Ethical Committee, we collected data from 44 patients who underwent haplo-identical HSCT at AFBMTC/NIBMT from the year 2015 to 2022. Written informed consent had already been taken at the time of HSCT from all cases for inclusion in ongoing and future studies. Diseases included in the study were acute myeloid leukemia (AML), acute lymphoid leukemia (ALL), myelodysplastic syndrome (MDS), lymphoma, aplastic anemia (AA), severe combined immunodeficiency (SCID), hemophagocytic lymphohistiocytosis (HLH) and X-linked agammaglobulinemia (XLA). These diseases were divided into three categories, i.e., BMF syndromes, HM, and Miscellaneous (Misc) group. BMF included all cases of AA, HM included AML, MDS, ALL, and Lymphomas while in the Misc group we included all cases of immune disorders like SCID, HLH, and XLA.

HLA class 1 and II antigens, determined by DNA-based low/intermediate resolution typing, were done for all recipients and donors. First-degree related haplo-identical donors, who were at least 5/10 HLA identical were selected for HSCT. Luminex Panel reactive antibody (PRA) panel and donor-specific antibodies (DSA) were done for all the donors. As per institutional policy, only DSA-negative donors were selected for haplo-HSCT.

All patients were admitted to isolation rooms with high-efficiency particulate air (HEPA) filters and laminar flow, following the institutional standard precautions at the time of their transplants. As per the institution’s infection control guidelines, neutropenic patients were managed as per the standard protocol. Nasal and axillary swab testing for screening of Methicillin-resistant Staphylococcus aureus (MRSA) was carried out for all patients along with the screening of carbapenamase-resistant enterococci (CRE) and vancomycin-resistant enterococci (VRE) in stool samples. Pre-transplant CMV Immunoglobulin G (IgG) status was checked in all recipients and donors. Antiviral, Pneumocystis jirovecii, and anti-fungal prophylaxis was given to all the patients. Quantitative CMV PCR testing was used to see the post-transplant CMV activation status of all patients at day +15 until day +100.

Both myeloablative and non-myeloablative conditioning regimens were used, depending upon the disease and indication of transplant. Conditioning regimens used are given in Table 1.

**Table 1:** Conditioning protocols used in different diseases Bu: Busulphan, Cy: Cyclophosphamide, Flu: Fludarabine, Mel: Melphalan, PtCy: Post Transplant Cyclophosphamide, ATG: Anti-Thymocyte Globulin, Ritx: Rituximab, TT: Thiotepa, Treo: Treosulfan, AML: Acute Myeloid Leukemia, ALL: Acute Lymphoid Leukemia, AA: Aplastic Anemia

S.No	Disease	Conditioning Regimen
	AML	Bu16,Cy100mg/kg with PTCy100 Flu150mg/m^2^,Bu12,Mel140 with PTCy100 Flu150 mg/m^2^,Bu12,Mel140,ATG10 with PTCy100 Flu150mg/m^2^,Bu12.8 with PTCy100 Flu150mg/m^2^,Bu9.6,Mel140 with PTCy100 Flu150mg/m^2^,Cy100mg/kg,Ritx375,TG7.5,Bu8 with PTCy100
	ALL	Flu150mg/m^2^,Bu9.6,Thiotepa10 with PTCy100 Flu150mg/m^2^,Bu9.6,Thiotepa10,ATG7.5 with PTCy100 Flu150mg/m^2^,Bu9.6,Mel140,ATG5 with PTCy100
	Lymphoma	Flu150mg/m^2^,Bu9.6,Thiotepa10,ATG7.5 with PTCy100
	AA	Flu120mg/m^2^,Cy100mg/kg, TG5 with PTCy100 Flu160mg/m^2^,Cy100mg/kg,TT10,TG5 with PTCy100 Flu150mg/m^2^,Cy100mg/kg,Bu8,ATG20 with PTCy100 Flu150mg/m^2^,Cy100mg/kg,Bu6.4,TG10 with PTCy100 Flu150mg/m^2^,Cy50mg/kg,Bu6.4,TG10 with PTCy100
	Miscellaneous	Flu160mg/m^2^,Bu10,Ritx375,TG7.5 with PTCy100 Flu30mg/m^2^,Bu10,TG7.5 with PTCy100 Flu1.2mg/m^2^,Treo32,TG7.5 with PTCy100 Flu180mg/m^2^,Bu12,TG7.5 with PTCy100

Patients were divided into two groups based on the type of GVHD prophylaxis used. Group 01 included those patients who received ATG/TG with PTCy100 while Group 02 included patients receiving only PTCy100.

Bone marrow harvest (BMH), peripheral blood stem cells (PBSC), or both (BMH+PBSC) were used as sources of stem cells, based on variables such as major ABO mismatch, weight difference between donors and recipients, donor choice, and target CD34 dose. Cyclosporine, mycophenolate mofetil, and post-transplant Cyclophosphamide were used as graft versus host disease (GVHD) prophylaxis. ATG was used depending on the indication of HSCT and GVHD risk. Lineage-specific chimerism was done at days +28, +100 and +180 for post-transplant monitoring. More frequent testing was done for patients with mixed donor chimerism or minimal residual disease (MRD) positivity. For pre-emptive CMV treatment (copies>2,000 copies/mL), Valgancyclovir and Ganciclovir were used and continued until two consecutive PCRs were negative. Leukodepleted and irradiated blood products were used for all the patients.

Definitions

Neutrophil engraftment was defined as the first day of an absolute neutrophil count of >0.5 x 10^9^/L for consecutive three days. Early and late engraftment was defined with a cutoff of 15 days post-transplant. Engraftment occurring at <15 days was defined as early while engraftment occurring after 15 days was included in late engraftment. Cytokine release syndrome (CRS) was defined as the temperature of > 98.6^o^F occurring within the first five days post-transplant in the absence of documented infection, with the rise of inflammatory markers like C-reactive protein (CRP).

Statistical analysis

Overall survival (OS) was calculated by including all the living cases at the last follow up from the date of transplant. Relapse/disease-free survival (RFS) was calculated by including all those cases who were disease-free and alive at the last follow-up. GVHD and relapse-free survival (GRFS) was calculated by including those cases who were GVHD-free, relapse/ disease free, and alive at the last evaluation day. For categorical variables, the chi-square test was used, while for continuous variables we used the Mann-Whitney U test and Student’s t-test. Cumulative incidences of the transplant complications were computed using the competing risk approach and Gray methods. To determine the significance of variables and to see their effect on survival, univariate and multivariate Cox regression analysis was used. Kaplan Meier method and curves were used to see the probability of RFS, GRFS, and OS. Survival curves were compared using the log-rank test.

## Results

Baseline characteristics

A total of 44 patients were enrolled in our study based on our inclusion criteria. The patient characteristics are summarized in Table 2. These 44 patients included 25 males and 19 females (male to female ratio, 1.3:1). Median age at transplant was 18 (1-39) years. Transplant indications included AA in 21 (47.7%) cases, 15 (34.1%) HM, and eight (18.2%) cases falling in the Misc group. The median time from the diagnosis of the disease to transplant was eight (5-16) months.

**Table 2:** Study characteristics of study population (n=44) AML: Acute Myeloid Leukemia, ALL: Acute Lymphoid Leukemia, SCID: Severe Combined Immunodeficiency, HLH: Hemophagocytic lymphohistiocytosis, XLA: X-linked agammaglobulinemia

Characteristics	Value
Patients	
Median age (years)	18 (1-39)
Gender (Male/Female)	25/19 (1.3:1)
Age group (years)	
0-10	14 (31.8%)
11-20	12 (27.3%)
21-30	12 (27.3%)
31-40	06 (13.6%)
Disease	
BMF (Aplastic anemia)	21 (47.7%)
Hematological malignancies	15 (34.1%)
AML	10
ALL	04
Lymphoma	01
Miscellaneous	08 (18.2%)
SCID	01
HLH	05
XLA	02
Donor	
Median age (years)	27 (18-33)
Gender (Male/Female)	28/16 (1.75:1)
Donor relationship	
Sibling	28 (63.6%)
Father	09 (20.4%)
Mother	07 (15.9%)
Gender mismatch	20 (45.4%)
ABO mismatch	
No	28 (63.6%)
Minor	10 (22.7%)
Major	03 (9.09%)
Bidirectional	03 (9.09%)
Time from diagnosis to transplant in months(IQR)	08 (5-16)

Transplant characteristics

Different conditioning protocols are used over time and are summarized in Table 1. Transplant characteristics of the study cohort are summarized in Table 3.

**Table 3:** Transplant characteristics of study population BM: Bone Marrow, PBSC: Peripheral blood stem cells, PTCy: Post transplant cyclophosphamide, ATG: anti-thymocyte globulin, PRA: Panel Reactive Antibodies, DSA: donor-specific alloantibody, CMV: Cytomegalovirus, TNC: Total nucleated cells

Source of stem cells	
BM	21 (47.7%)
BM+PBSC	18 (40.9%)
PBSC	05 (11.4%)
Conditioning type	
PTCy alone	09 (20.4%)
PTCy with ATG/TG	35 (79.5%)
Luminex PRA (+/-)	02/42
DSA (+/-)	-/44
Pre-transplant CMV-IgG status (Patient)	
Positive	38 (86.4%)
Negative	06 (13.6%)
CMV IgG-status (Donor)	
Positive	41 (93.1%)
Negative	03 (6.81%)
CD34 dose, x 10^6^/kg, Median(IQR)	6.51 (2.1-20.3)
BM	6.09 (2.4-13.4)
BM+PBSC	7.22 (2.1-20.3)
PBSC	6.03 (4.7-7.3)
CD34 dose, x 10^6^/kg	
<5	11 (25%)
>5	29 (65.9%)
Missing data	04
TNC dose, x 10^8^/kg,Median	8.78 (2.5-18.8)
Engraftment (yes/No)	34/10 (77%)
Early	16 (47%)
Late	18 (53%)
PGF	10 (22.7%)
Neutrophil engraftment-days Median(IQR)	16 (14-17)
Median hospital stay (days)	36 (15-84)

BMH was used in 21 (47.7%) cases, PBSC was used in five (11.4%) cases while BMH+PBSC was used in 18 (40.9%) cases as a source of stem cells. Successful engraftment was achieved in 34 (77.2%) cases. Primary graft failure (PGF) occurred in 10 (22.7%) cases while secondary graft failure occurred in four (9.1%) cases. Transplant complications are summarized in Tables 4, 5. Maximum number of graft failures 11 (25%) occurred in BMF group (p-value=0.04). Out of 44 cases, nine (20.5%) had aGVHD while 10 (22.7%) had cGVHD and only two (4.5%) cases of overlap syndrome were found in our study population. Among the disease categories, the greatest incidence of aGVHD four (26.6%) was found in HM while maximum cases of cGVHD five (50%) were found in BMF group (p-value=0.87). CRS occurred in 28 (63.6%) of cases while only three (6.8%) had VOD. Disease relapse occurred in four (9.1%) of the study population. CMV reactivations occurred in 31 (70.5%) cases, BKV virus activation occurred in nine (20.5%) cases with hemorrhagic cystitis occurring in seven (15.9%). Only one (2.3%) case had Epstein-Barr virus (EBV) reactivation. Association of various risk factors and outcomes with conditioning types is summarized in Table 6.

**Table 4:** Transplant complications GVHD: Graft versus host disease, CMV: Cytomegalovirus, EBV: Epstein-Barr virus, VOD: Veno-occlusive disease, CRS: Cytokine release syndrome

Complication	Cases n (%)
Relapse	04 (9.09%)
Graft Failure	14 (31.8%)
Primary	10 (22.7%)
Secondary	04 (9.1%)
GVHD Acute	09 (20.5%)
Grade-II	05
Grade-III	03
Grade-IV	01
Chronic	10 (22.7%)
Limited	06
Extensive	04
Overlap Syndrome	02 (4.5%)
CMV Reactivations	31 (70.5%)
Other Viral reactivations BKV	09 (20.4%)
EBV	01 (2.2%)
Hemorrhagic Cystitis	07 (15.9%)
VOD	03 (6.8%)
CRS	28 (63.6%)
Median Follow-up time (months)	09 (01-36)
Overall Mortality	22 (50%)
Transplant related mortality	17 (77.2%)
Non transplant related mortality	05 (22.7%)

**Table 5:** Disease vise distribution of complications BMF: Bone marrow failure, HM: Hematological malignancies, GVHD: Graft versus host disease, VOD: Veno-occlusive disease, CRS: Cytokine release syndrome, CMV: Cytomegalovirus, EBV: Epstein-Barr virus, TRM: Transplant related mortality

Complications	Cumulative incidence with 95% confidence interval (%)						
	BMF (21) n, %(CI)		HM (15) n, %(CI)		Miscellaneous (8) n, %(CI)		P-value
GVHD							0.87
Acute	3	14.3(4.9-34.6)	4	26.6(10.9-51.9)	2	25(71.5-59)	
Chronic	5	23.8(10.6-45)	4	26.6(10.9-51.9)	1	12.5(2.2-47)	
Overlap	1	4.7(0.8-22.6)	1	6.6(1.1-29.8)	0	0	
Graft Failure							0.04
Primary	7	33.3(17.1-54.6)	2	13.3(3.7-37.8)	1 12.5(2.2-47)		
Secondary	4	19(7.6-40)	0	0	0	0	
CMV reactivations	15	71.4(50-86.1)	13	86.6(62.1-96.2)	03	37.5(13.6-69.4)	0.04
Relapse	1	4.7(0.8-22.6)	3	20(7-45.1)	0	0	0.17
VOD	1	4.7(0.8-22.6)	1	6.6(1.1-29.8)	1	12.5(2.2-47)	0.76
CRS	15	71.4(50-86.1)	8	53.3(30.1-75.1)	5	62.5(30.5-86.3)	0.53
Other viral activations							0.13
BKV	6	28.5(13.8-49.9)	3	20(70.5-45.1)	0	0	
EBV	0	0	0	0	1	12.5(2.2-47)	
TRM	10	47.6(28.3-67.6)	5	33.3(15.1-58.2)	2	25(71.5-59)	0.74

**Table 6:** Association of risk factors and outcomes with conditioning type (PTCy alone/ PtCy+ATG) GVHD: Graft versus host disease, VOD: Veno-occlusive disease, CRS: Cytokine release syndrome, CMV: Cytomegalovirus, EBV: Ebstein-Barr Virus, ATG: anti-thymocyte globulin, PTCy: Post-transplant Cyclophosphamide

Risk Factors	ATG+PTCy, n=35	PTCy, n=9	Total, n=44	P-value
VOD	02	01	03	0.567
GVHD				0.10
Acute	8	1	09	
Chronic	6	4	10	
Overlap	1	1	02	
CRS	24	04	28	0.18
Relapse	2	2	04	0.12
Graft Failure	12	01	13	0.160
Type of graft failure				0.33
Primary	9	1	10	
Secondary	4	0	04	
Neutrophil Engraftment				0.07
Early	10	06	16	
Late	16	02	18	
CMV Viral activation	23	08	31	0.17
Other Viral activations				0.626
BKV	8	01	09	
EBV	01	00	01	

Survival

**Figure 1:**
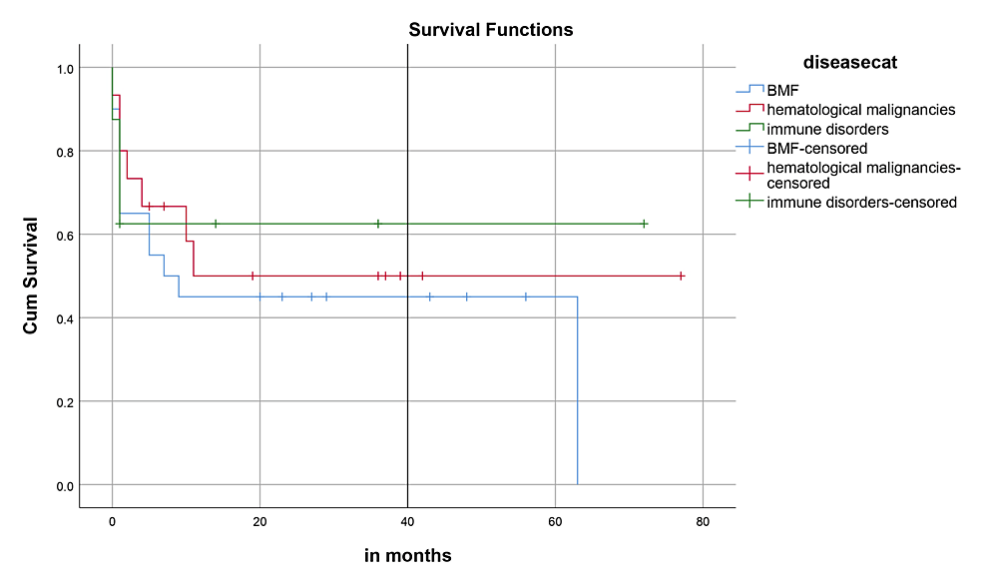
Effect of disease: OS was 40%,53.3% and 62.5% in BMF, HM, and Miscellaneous group (p-value=0.65) OS: Overall survival, BMF: Bone marrow failure, HM: Hematological malignancies

**Figure 2:**
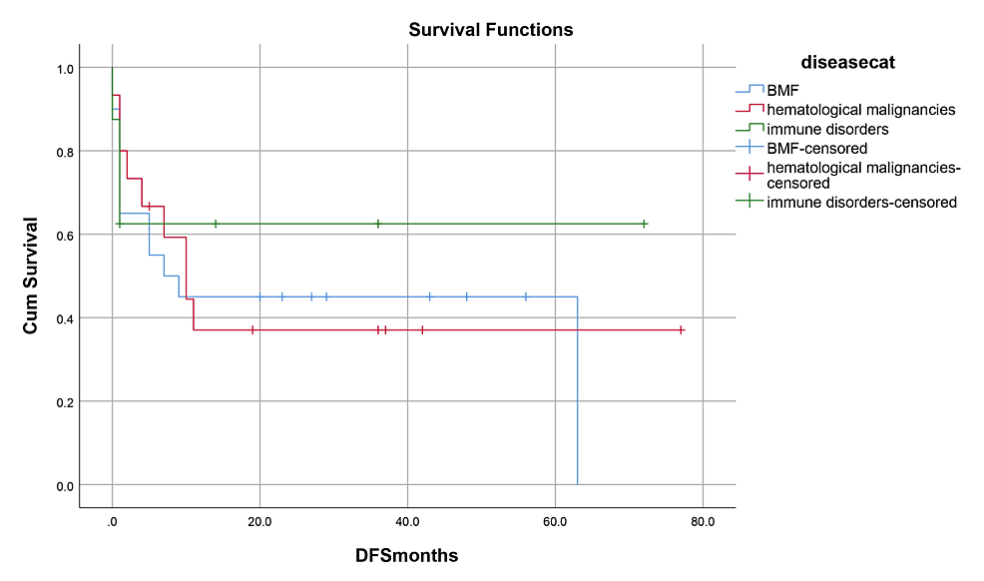
Effect of disease: RFS was 40%, 40%, and 62.5% in BMF, HM, and Miscellaneous group (p-value=0.73) RFS: Relapse-free survival, BMF: Bone marrow failure, HM: Hematological malignancies

**Figure 3:**
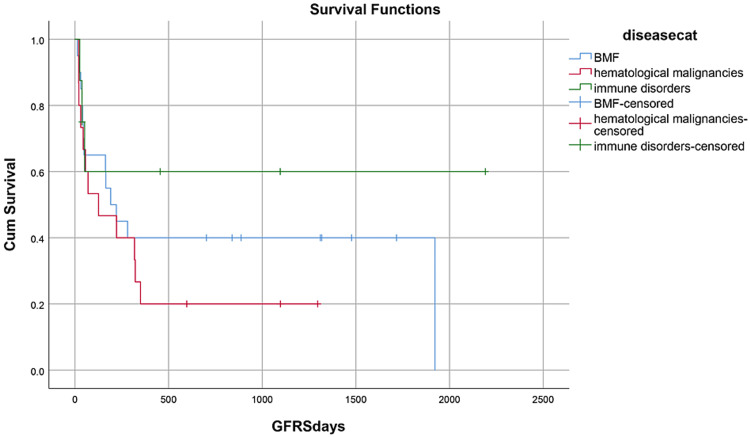
Effect of disease: GRFS was 35%, 20%, and 62.5% in BMF, HM, and Miscellaneous group (p-value=0.29) GRFS: GVHD-free relapse-free survival, BMF: Bone marrow failure, HM: Hematological malignancies

**Figure 4:**
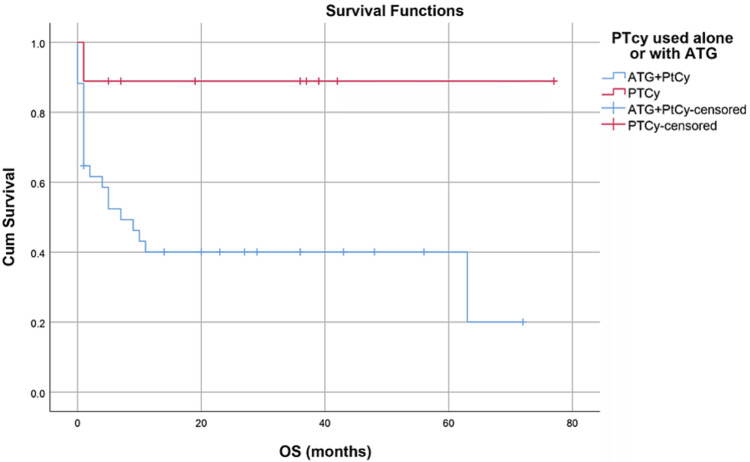
Conditioning regimen: OS was 38.2% in ATG+PTCy and 88.9% in PTCy group (p-value=0.018) OS: Overall survival, ATG: Anti-thymocyte globulin, PTCy: Post-transplant cyclophosphamide

**Figure 5:**
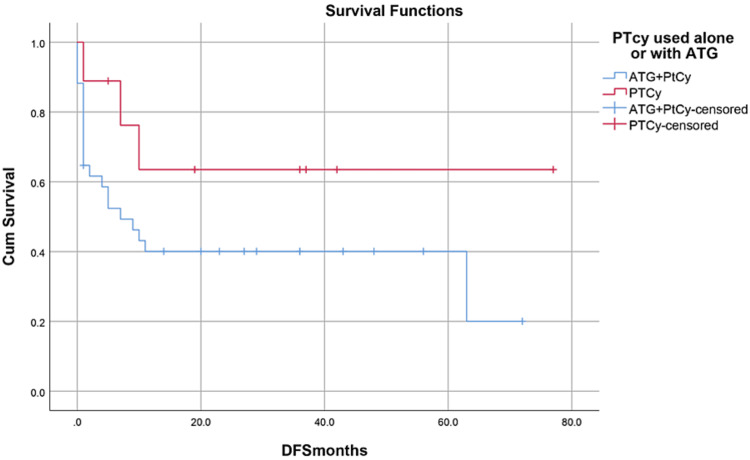
Conditioning regimen: RFS was 38.2% in ATG+PTCy and 68.7% in PTCy group (p-value=0.126) RFS: Relapse-free survival, ATG: Anti-thymocyte globulin, PTCy: Post-transplant cyclophosphamide

**Figure 6:**
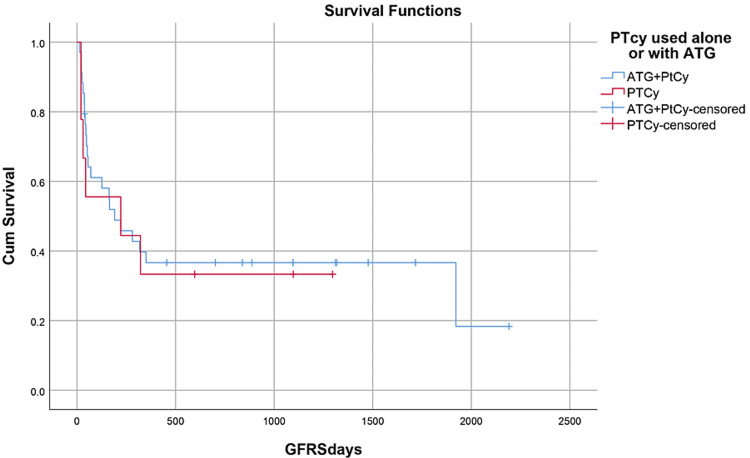
Conditioning regimen: GRFS was 35.3% in ATG+PTCy and 33.3% in PTCy group (p-value=0.763) GRFS: GVHD-free relapse-free survival, ATG: Anti-thymocyte globulin, PTCy: Post-transplant cyclophosphamide

Median hospital stay in our patients was 36 (15-84) days while median follow-up time was nine months (IQR, 1-36). Median hospital stay of patients with BMF was 35 (15-70) days, HM 46 (28-84) days, and while in Misc group; it was 41 (28-62) days. However, there was no statistically significant difference between them. Within the first 100 days, 14 patients died with an overall 100-day mortality of 31.8% while if we categorize it into the disease categories, BMF had a 100-day mortality rate of seven (33.3%), in HM it was four (26.6%) while in Misc group it was 3(37.5%). OS, relapse-free survival (RFS) and GVHD-free relapse-free survival (GRFS) of our study population was 50%, 45.4%, and 36.3%, respectively. OS in BMF, HM and Misc groups (Figures 1-3) was 40%, 53.3% and 62.5%, respectively (p-value 0.65). OS, RFS and GRFS in patients who received ATG+PTCy (Figures 4-6) conditioning regimen was 38.2%, 38.2%, 35.3% respectively while in those cases who received PTCy only was 88.9%,68.7% and 33.3%, respectively. There was a statistically significant difference (p-value=0.018) in the OS of group receiving ATG+PTCy conditioning versus those receiving PTCy alone.

 

**Figure 7:**
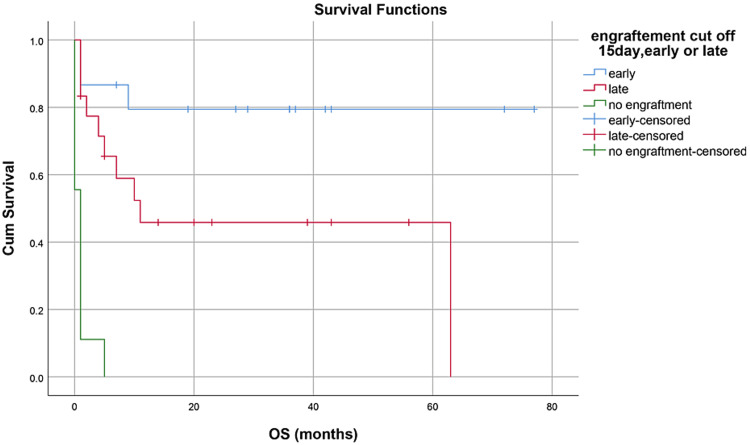
Neutrophil engraftment: OS was 80% in early group, 44.4% in late group, and 0% in PGF group (p-value=0.000)

**Figure 8:**
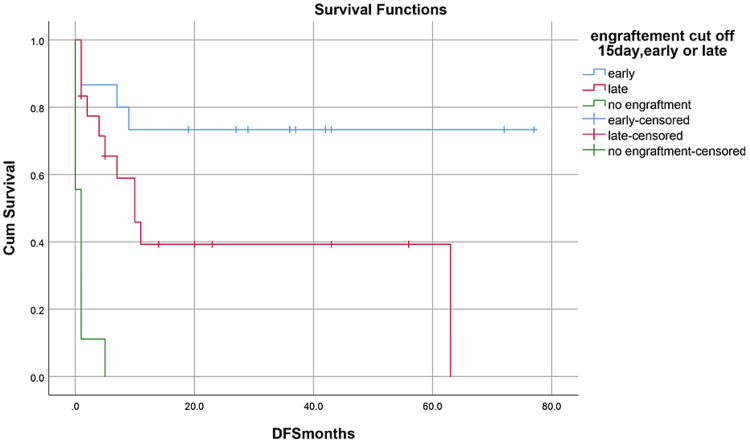
Neutrophil engraftment: RFS was 73.3% in early group, 38.9% in late group, and 0% in PGF group (p-value=0.00)

**Figure 9:**
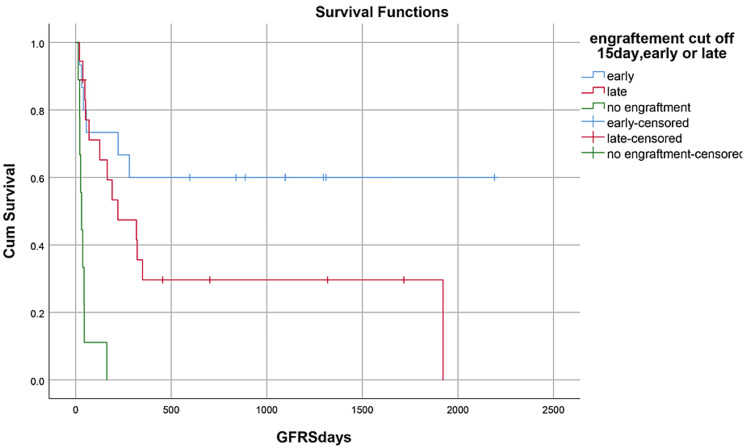
Neutrophil engraftment: GRFS was 60% in early group, 27.8% in late group, and 0% in PGF group (p-value=0.00)

**Figure 10:**
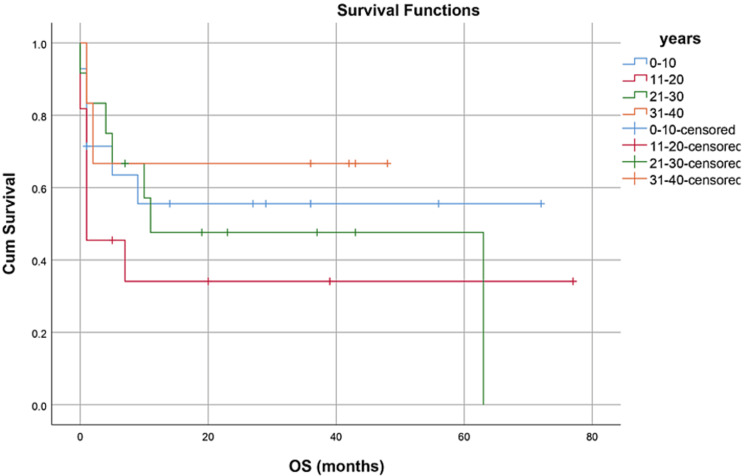
Age groups: OS was 57.1%, 36.4%, 41.7%, 66.7% (p-value=0.556)

**Figure 11:**
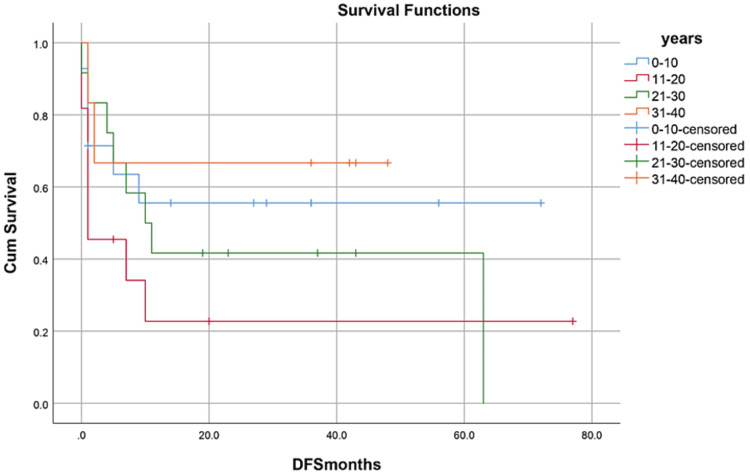
Age groups: RFS was 57.1%, 27.3%, 33.3%, 66.7% (p-value=0.318)

**Figure 12:**
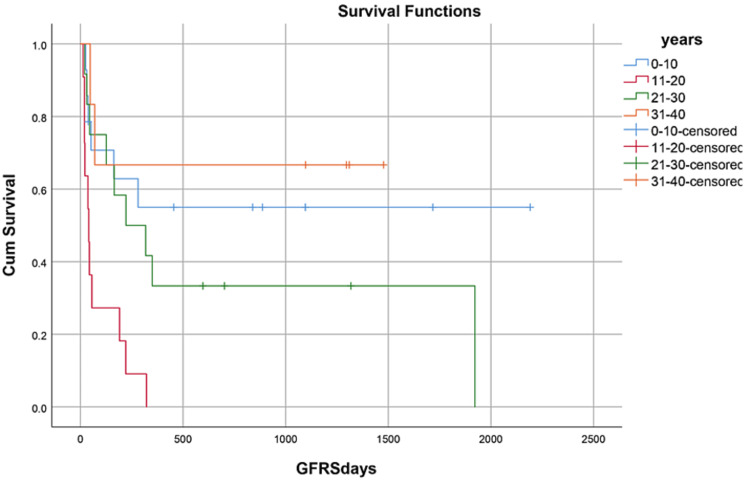
Age groups: GRFS was 57.1%, 0%, 25%, 66.7% (p-value=0.001)

**Figure 13:**
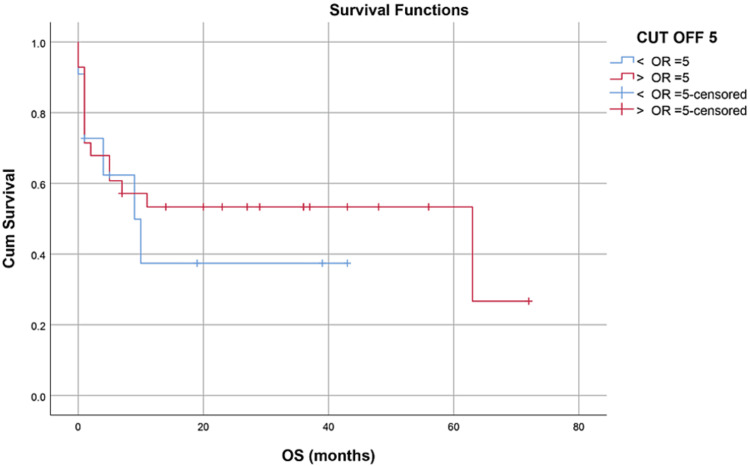
Effect of CD34 dose: OS was 45.5% in >5 CD34 group, 50% in <5 CD34 group (p-value=0.554)

**Figure 14:**
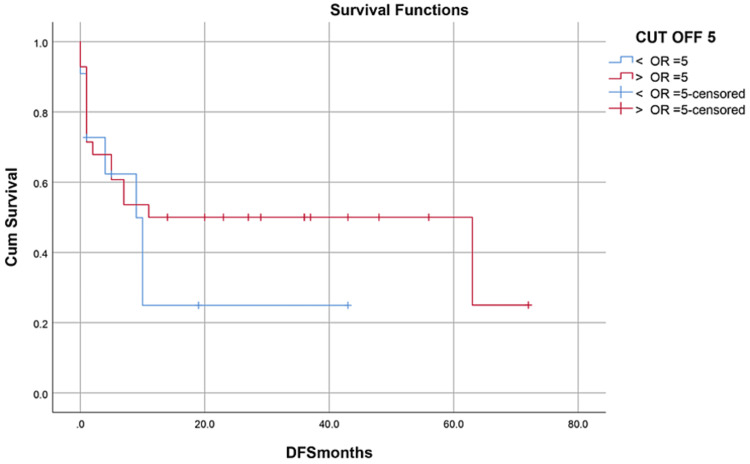
Effect of CD34 dose: RFS was 36.4% in >5 CD34 group 46.4% in <5 CD34 group (p-value=0.175)

**Figure 15:**
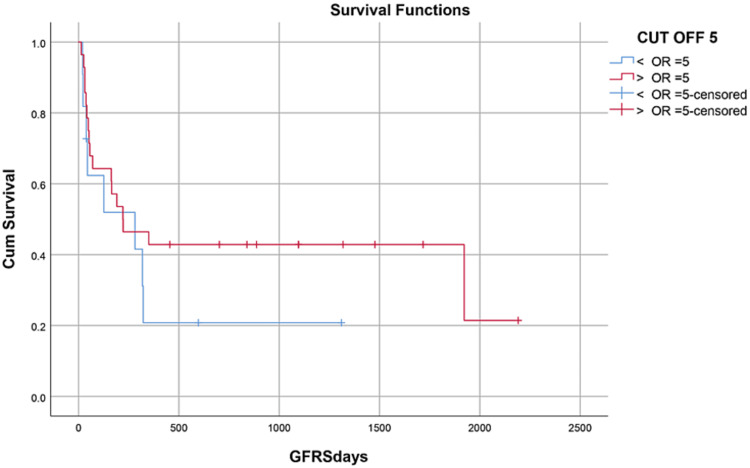
Effect of CD34 dose: GRFS was 27.3% in >5 CD34 group, 39.3% in <5 CD34 group (p-value=0.298)

**Figure 16:**
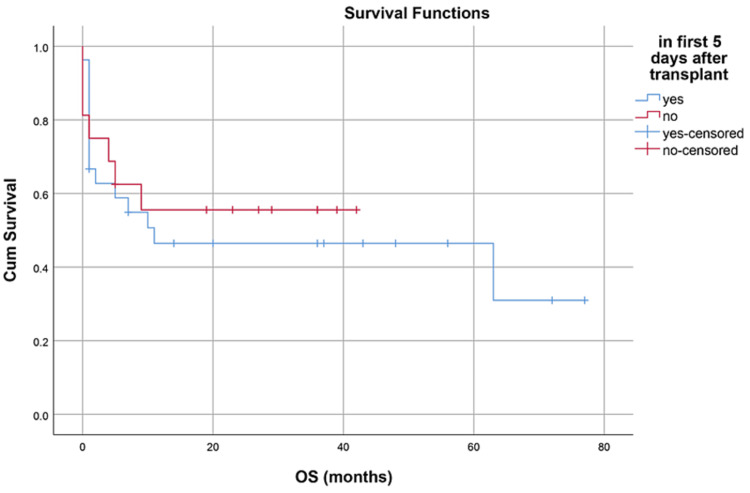
Effect of CRS: OS was 44.4% in CRS group, 56.3% in non-CRS group (p-value=0.733) CRS: Cytokine-release syndrome

**Figure 17:**
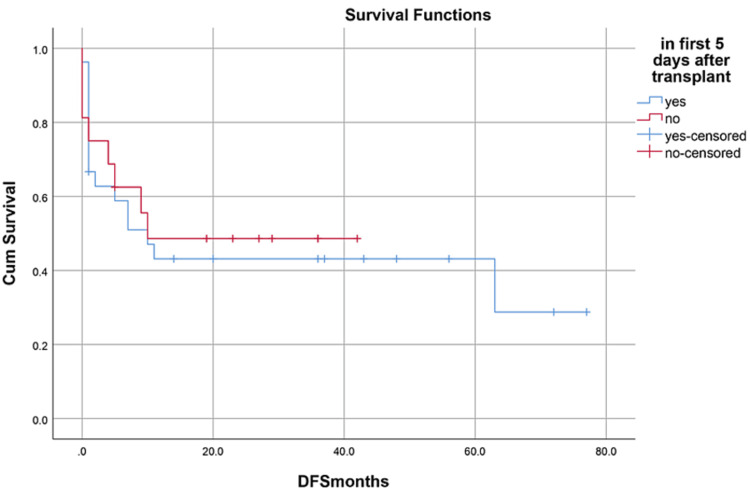
Effect of CRS: RFS was 40.7% in CRS group, 50% in non-CRS group (p-value=0.842) CRS: Cytokine-release syndrome

**Figure 18:**
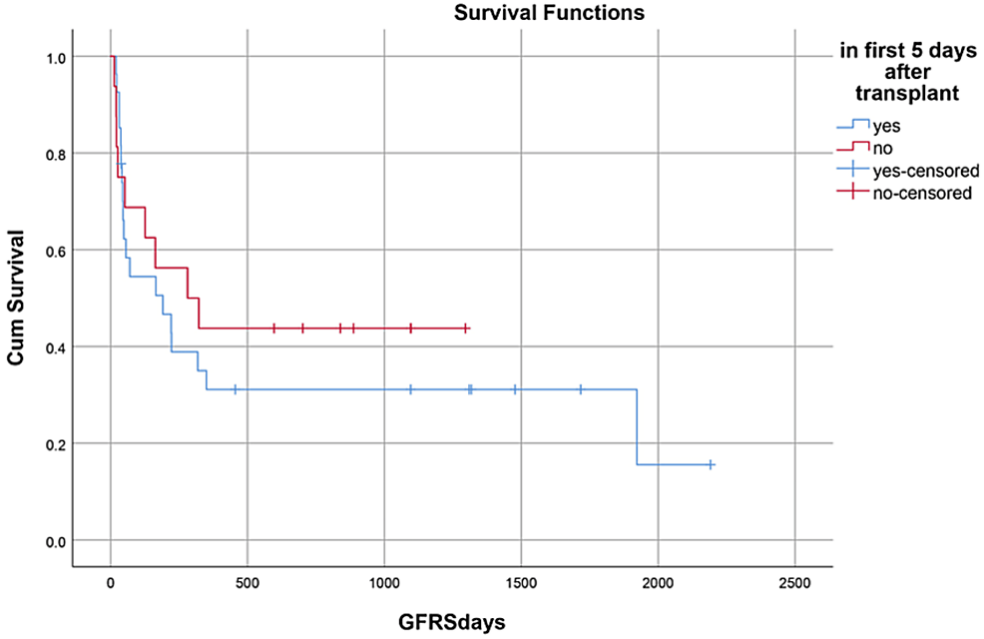
Effect of CRS: GRFS was 29.6% in CRS group, 43.8% in non-CRS group (p-value=0.513) CRS: Cytokine-release syndrome

OS, RFS, and GRFS in early neutrophil engraftment group was 80%, 73.3%, and 60% while in late engraftment group it was 44.4%, 38.9%, and 27.8%, respectively (Figures 7-9). Early engraftment group had a superior OS, RFS, and GRFS (p-value=0.000) than those with late engraftment. A statistically significant difference was found in the GRFS (p-value=0.001) in different age groups (Figures 10-12). Best outcomes in terms of OS, DFS, and GRFS were found in 31-40 years of age group (66.7%). There was no statistically significant difference in OS, or DFS for patients receiving less or more than 5x10(6)/kg CD34 dose (p-value=0.21) (Figures 13-15). In patients developing CRS, OS, RFS, and GRFS were 44.4%, 40.7%, and 29.6%, respectively, while those who did not develop it had an OS of 56.3%, RFS 50%, and GRFS 43.8% (Figures 16-18).

Overall, 22 (50%) patients died post-transplant. Among these, 17 cases (38.6%) had transplant-related mortality (TRM) while five (11.4%) had non-transplant-related mortality. TRM included deaths due to primary graft failure seven (41.1%), secondary graft failure one (5.8%), GVHD three (17.6%), infections and sepsis three (17.6%), relapse one (5.8%) and other causes like multiorgan failure, hepatotoxicity and intracranial bleed two (11.7%). On multivariate Cox regression analysis, conditioning type, neutrophil engraftment (early/late), and GVHD had a statistically significant association with OS, while age of the patient, ABO and gender mismatch, disease type, graft failure, relapse, VOD, CRS, source of stem cells, CD-34 dose, and viral activations had no effect on OS (Table 7).

**Table 7:** Association of risk factors with overall survival-multivariate Cox regression analysis GVHD: Graft versus host disease, CRS: Cytokine-release syndrome, VOD: Veno-occlusive disease, CMV: Cytomegalovirus, TNC: Total nucleated cells

Risk factors	HR(95% CI)	P-value
Overall Survival(OS)		
Age of patient	1.009(0.95-1.07)	0.75
ABO mismatch	1.35(0.88-2.08)	0.15
Gender mismatch	0.92(0.39-2.17)	0.85
Time to transplant	0.99(0.96-1.03)	0.95
Disease	3.23(0.5-20.8)	0.21
Conditioning	0.13(0.18-1.03)	0.05
Source of stem cells	0.55(0.23-1.32)	0.18
TNC dose	0.043(0.85-1.07)	0.46
CD34 dose	0.74(0.18-3.06)	0.68
CRS	0.61(0.21-1.78)	0.36
Neutrophil engraftment (Early/ Late)	15.7(0.95-260)	0.05
GVHD	0.15(0.12-0.91)	0.04
Graft failure	0.06(0.00-2.16)	0.12
Relapse	1.3(0.25-6.74)	0.74
VOD	0.41(0.07-2.24)	0.30
CRS	0.61(0.21-1.78)	0.36
CMV activation	1.90(0.57-6.3)	0.29
Other viral activations	0.73(0.35-1.54)	0.42

## Discussion

This retrospective study was carried out at a tertiary care center in resource-limited settings. While the data suggests a rising global trend towards haplo-identical HSCT [8], it poses unique difficulties and challenges in a developing country like Pakistan. These challenges are multifactorial and stem from the non-availability of an HLA registry at the national level, non-availability of high-resolution HLA typing, underdeveloped healthcare infrastructure lacking adequate supportive paraphernalia, high prevalence of CMV Seropositivity in the general population, higher prevalence of Multi-Drug Resistant bugs, non-availability of newer transplant techniques like T-Cell depleted grafts, non-availability of better antivirals and antimicrobials, chronically malnourished patients at time of transplant, etc.

Our center started doing Haplo-Identical HSCT for various hematological disorders in 2015. We have carried out 44 Haplo-HSCT, till the date of study. The median age of patients in our study group was 18 years, which is similar to regional data from the neighboring country [10]. However, data published by Arcuri et al. [11] and by Fuchs et al. [12], describe patients with older median age at Haplo-transplant, Plausible reasons for this could be better health care services allowing earlier diagnosis, adequate disease control by attaining deeper remissions and disease surveillance, ultimately leading to a delay in transplant. The major indication for Haplo-Identical HSCT in our study was AA (AA) in 21 (47.7%) cases followed by HM in 15 (34.1%). On the contrary, regional and Western data suggest that the most common indication for Haplo-identical HSCT was AML [13]. The majority of the donors in our study were Haplo-matched siblings, followed by fathers and mothers 28 (63.6%), nine (20.4%), and seven (15.9%), respectively. This finding is in keeping with data published by DeZern et al. [14].

The source of stem cells was BM in 21 (47.7%) cases, BM + PBSC in 18 (40.9%), and PBSC alone in five (11.4%) cases, yielding a median CD34+ dose of 6.51 x 10^6^/kg. OS, RFS, and GRFS were superior in patients receiving a CD34+ dose of more than 5.0 x 10^6^/kg as compared to lower doses, in keeping with a study published by Maffini et al. [15]. This finding needs further research to establish statistical significance. Neutrophil engraftment took place on a median day of +16 in our study. OS, RFS, and GRFS were significantly higher (p-value=0.000) in cases where early neutrophil engraftment took place (80%, 73.3%, and 60%), as compared to cases with late engraftment (44.4%, 38.9% and 27.8%). Ten (22.7%) cases that did not achieve engraftment suffered transplant-related mortality (TRM). There is a paucity of regional as well as international studies, which have compared the effect of early vs late neutrophil engraftment on OS, RFS, and GRFS in haplo-Identical HSCT.

The median hospital stay of our patients was 36 days. This prolonged stay is attributable to several factors like late engraftment, primary graft failure, nosocomial infections, and related complications, etc. It is interesting to point out that studies like a single center experience from Mexico, published by Pedraza et al. [16] describe how their center has developed an outpatient haplo-HSCT program and was not only able to shorten the median hospital stay to eight days, but also cut down the cost of transplant effectively while yielding acceptable results.

Our study also showed that GFRS was higher (67%) in the age group of 31-40 years (p-value=0.001) as compared to other age groups. However, data published by Byung-Sik et al. [17] showed that GRFS approaches 41% for haplo-HSCT in AML, but the age has no significant effect. The OS was higher (62.5%) in the Misc group as compared to the other disease groups in our study; however, it was not statistically significant.

All patients and their donors were tested for CMV during pre-transplant assessments. CMV-IgG status was positive in 38 (86.4%) patients (pre-transplant) and CMV-IgG was positive in 41 (93.1%) of donors. This high percentage is in keeping with the high regional sero-prevalence of CMV in the general population [18]. None of our patients or donors received prophylactic antivirals. Various complications encountered during haplo-identical transplants are tabulated in Table 5. Among them, CMV reactivation and graft failure had statistically significant associations with different disease categories. CMV reactivation was encountered in 70.5% of the cases, with maximum cases (86.6%) from the HM group. CMV reactivation in our study was higher, when compared with data published by Ramya et al. [19], who have reported an incidence of 43.7% in a tertiary care center in South India. Interestingly, we had 20.4% BKV reactivations, which only occurred as a co-infection with CMV. Out of these, seven (15.9%) patients suffered from BKV-related hemorrhagic cystitis. Kerbauy et al. [20], in a study on haplo-HSCT cases, have reported CMV+BKV coinfection in 38.9% of cases, with BKV-related HC in 30.6% of cases, which is higher as compared to our study. In our study, conditioning type was not a risk factor for CMV reactivation. All of these reactivations occurred before Day + 100. Patients with CMV titers > 2,000 IU/mL were treated with Valgancyclovir at 900mg twice daily with weekly monitoring of CMV titers and blood counts. Treatment was stopped when CMV titers receded to less than 2,000 IU/mL if blood counts dropped or if the patient developed drug-related side effects. CMV reactivation, however, did not have a statistically significant effect on OS. In a study published by Lin et al. [21], there was a higher cumulative incidence of CMV infection necessitating therapy in patients not receiving Letermovir (68.8% (95% CI, 48.9- 82.2)) when compared with those receiving prophylaxis (21.9% (95% CI, 9.5-37.6)). This study also showed that CMV reactivation had no effect on the OS, and our study has yielded similar results. Our institution ensures a strict policy on post-transplant CMV testing and prompt treatment, which is believed to be the cause of these comparable results despite a higher incidence of reactivations. Nonetheless, CMV reactivation poses a formidable challenge in our settings, considering financial constraints, the non-availability of better antivirals, and the logistic difficulties our post-transplant patients face for regular follow-ups. Letermovir is not available in Pakistan, and its use can potentially improve the post-transplant quality of life in our patients.

The incidence of graft failure (31.8%) in our study was higher as compared to results published by Poudyal et al. [22] on a larger cohort of 66 cases. We believe that the incidence of GF may rise with a larger cohort, considering various variables like higher sero-prevalence of CMV and a higher incidence of CMV reactivations, use of Valgancyclovir, higher incidence of sepsis, more secondary graft failures, etc. In our study, primary graft failure occurred in 22.7% and secondary graft failure in 9.1% cases, which was significantly higher as compared to data published in a study from a neighboring country by George et al. [23]. The higher incidence of graft failures in our study can be attributed to the non-availability of TBI at our center. It is also important to note that graft failure had no statistically significant effect on OS in our study, in contrast to findings published by George et al. [23].

The incidence of aGVHD was 20.5% (Grades II-IV), and cGVHD was 22.7% (Limited-Extensive). Overlap syndrome had an incidence of 4.5%. Incidence of GVHD was 44.1% in ATG + PTCy arm and it was higher at 66.7% in PTCy alone arm. Our results of the PTCy+ATG arm are in keeping with data published by Barkhordar et al. [24].

Our results also showed a statistically significant relation between the type of conditioning and OS, in keeping with data published by George et al. [23]. Overall survival was significantly higher (p-value=0.018) in PTCy alone arm (88.9%) when compared with ATG+PTCy arm (38.2%) in our study. These results can be attributed to a higher occurrence of graft failure and more cases of late neutrophil engraftment in the ATG+PTCy arm in our study.

Our study was limited to a single center, which may have affected the reliability and representativeness of the results for our region. To achieve greater reliability and a broader representation, a collaborative multicenter study with a larger patient cohort would have been ideal. However, due to the mentioned challenges, haplo-identical transplants are infrequently performed, if at all, in most centers across Pakistan.

## Conclusions

The findings from the current study emphasize that haplo-identical HSCT is a complex decision that demands specialized expertise. Several factors contribute to its challenges, including the absence of advanced techniques like T-cell depleted allografts, non-availability of CMV anti-viral agent Letermovir, resource constraints, scarcity of regional data, and developing healthcare infrastructure, all of which present multiple hurdles.

To achieve comparable results to well-established centers, it is crucial to address these variables. By implementing newer techniques, securing access to essential medications, bolstering resources, collecting comprehensive regional data, and further developing our healthcare infrastructure, we can improve the outcomes of haplo-identical HSCT and provide better care for our patients.
